# A Systematic Study of Ammonia Recovery from Anaerobic Digestate Using Membrane-Based Separation

**DOI:** 10.3390/membranes12010019

**Published:** 2021-12-24

**Authors:** Fanny Rivera, Raúl Muñoz, Pedro Prádanos, Antonio Hernández, Laura Palacio

**Affiliations:** 1 Institute of Sustainable Processes, University of Valladolid, 47011 Valladolid, Spain; fannymaritza.rivera@uva.es (F.R.); mutora@iq.uva.es (R.M.); ppradanos@uva.es (P.P.); antonio.hernandez@uva.es (A.H.); 2 Department of Applied Physics, Science Faculty, University of Valladolid, 47011 Valladolid, Spain; 3 Department of Chemical Engineering and Environmental Technology, University of Valladolid, 47011 Valladolid, Spain

**Keywords:** ammonia recovery, anaerobic digestate, flat sheet membranes, mass transfer, membrane fouling

## Abstract

Ammonia recovery from synthetic and real anaerobic digestates was accomplished using hydrophobic flat sheet membranes operated with H_2_SO_4_ solutions to convert ammonia into ammonium sulphate. The influence of the membrane material, flow rate (0.007, 0.015, 0.030 and 0.045 m^3^ h^−1^) and pH (7.6, 8.9, 10 and 11) of the digestate on ammonia recovery was investigated. The process was carried out with a flat sheet configuration at a temperature of 35 °C and with a 1 M, or 0.005 M, H_2_SO_4_ solution on the other side of the membrane. Polytetrafluoroethylene membranes with a nominal pore radius of 0.22 µm provided ammonia recoveries from synthetic and real digestates of 84.6% ± 1.0% and 71.6% ± 0.3%, respectively, for a membrane area of 8.6 × 10^−4^ m^2^ and a reservoir volume of 0.5 L, in 3.5 h with a 1 M H_2_SO_4_ solution and a recirculation flow on the feed side of the membrane of 0.030 m^3^ h^−1^. NH_3_ recovery followed first order kinetics and was faster at higher pHs of the H_2_SO_4_ solution and recirculation flow rate on the membrane feed side. Fouling resulted in changes in membrane surface morphology and pore size, which were confirmed by Atomic Force Microscopy and Air Displacement Porometry.

## 1. Introduction

A change in the perception of the uses of anaerobic digestion has occurred over past decades. Thus, anaerobic digestion was initially considered as a cost-competitive technology for organic matter stabilization in wastewaters and solid waste, then a sustainable platform for renewable electricity and heat generation via biogas production, and more recently, it has been regarded as the potential core of a multiproduct biorefinery. Energy and carbon in the form of biogas, along with nutrients dissolved in anaerobic effluents (typically called digestates), represent nowadays the main by-products from anaerobic waste treatment. NH_3_ recovery from digestates will prevent the pollution of natural water bodies, which is desirable as this nitrogenous compound is toxic to fish, increases oxygen demand and induces eutrophication [1]. NH_3_ is also harmful to humans as it causes respiratory problems and is considered a precursor of N_2_O, a potent greenhouse gas [2,3,4]. Therefore, NH_3_ recovery from digestates is crucial to improve the environmental sustainability of anaerobic digestion processes and could bring additional economic benefits. Unfortunately, only 10% of the nitrogen present in wastewater is recovered by conventional wastewater treatment plants (WWTPs) [5,6,7]. Today, there is a wide range of commercial technologies to remove ammonia from wastewater, but most of them entail high operating costs [8] and environmental impacts resulting from nitrogen conversion and release to the atmosphere. Thus, conventional physical/chemical technologies are based on selective ion exchange, air stripping, chemical precipitation, adsorption, advanced oxidation processes, breakpoint chlorination, etc. [9,10,11,12]. For instance, NH_3_ stripping ranks among the most popular, but energy demanding, technologies available to remove nitrogen from high-strength wastewater, with an average energy consumption of 4 kwh per kg of nitrogen [13]. On the other hand, conventional nitrogen removal in domestic WWTPs relies on nitrification, which is the conversion of ammoniacal nitrogen (NH_3_-N) to ${\mathrm{NO}}_{3}^{-}$, and denitrification, which is the conversion of ${\mathrm{NO}}_{3}^{-}$ to N_2_ gas [14,15,16]. In this context, 1.5 kWh of electricity are typically required to remove 1 kg of N in denitrification/nitrification or anammox processes [17]. In addition, conventional biological nitrogen removal in WWTPs entails 0.9 kg CO_2 eq_ per m^3^ of treated water [18,19]. Therefore, there is an urgent need to develop and implement cost-competitive and sustainable NH_3_ recovery technologies in high strength wastewater such as digestates [20,21,22].

Recently, the use of membranes for NH_3_ recovery from high strength wastewater has been proposed as a cost-competitive and environmentally friendly approach to upgrade residual nitrogen. For example, Brennan et al. [23] made an extensive analysis of costs demonstrating the feasibility of NH_3_ recovery by membranes over conventional methods at a pilot plant scale. Membrane contactors require less energy because of their high specific surface area, which provides a faster NH_3_ separation. In this context, membrane contactors have been successfully implemented for NH_3_ recovery from gaseous emissions [24,25,26]. Membrane contactors have also been implemented to support a direct NH_3_ recovery from wastewater [27,28,29]. This approach is based on hydrophobic membranes made of polymers highly permeable to NH_3_, which is transported through the membrane pores into the receiving liquid phase recirculating through the permeate side [20,21,30]. This permeating NH_3_ reacts with an acid contained in the permeate side of the membrane. For the process to be efficient, ammoniacal nitrogen has to be present in its volatile form (NH_3_), which can be ensured by increasing pH and temperature [22]. Ammonium sulphate, a commercial chemical fertilizer, can be generated during membrane-based NH_3_ recovery when using H_2_SO_4_ to boost NH_3_ diffusion (Equation (1)) [31]. The use of H_3_PO_4_ and HNO_3_ on the permeate side also results in the generation of chemical fertilizers such as ammonium phosphate and ammonium nitrate, respectively [27,28,29]. In this regard, Damtie and co-workers concluded that H_2_SO_4_ mediates a more effective ammonia capture than H_3_PO_4_ and HNO_3_ [32].

(1) $$2{\mathrm{NH}}_{3}+{\;H}_{2}{\mathrm{SO}}_{4}\;\to {\left({\mathrm{NH}}_{4}\right)}_{2}{\mathrm{SO}}_{4}$$

In this work, the performance of commercial flat sheet membranes for NH_3_ recovery from synthetic and real digestates was systematically evaluated. To optimize the operation conditions, the influence of digestate pH, within the range of coexisting NH_4_^+^, NH_3_ [33], and recirculation flow rate, was investigated using different concentrations of sulfuric acid on the other side of the membrane. To make this new ammonia process accessible, our proposal involves the use of commercial membranes that were previously employed for other applications. By scanning the variables that can be controlled at the place of installation, we reached the optimal conditions, without using the latest generation and non-commercial materials. Ultimately, our objective was to propose a solution within the reach of the farmer who intends to implement it in their facilities. Finally, membrane fouling was studied using Atomic Force Microscopy (AFM) to examine the surface morphology of the membranes and Air Displacement Porometry (ADP) to determine the corresponding changes of the pore sizes of the membranes.

## 2. Materials and Methods

### 2.1. Real and Synthetic Digestate Characterization

The synthetic digestate (SD) composition mimicked a real digestate and consisted of 5.0 g NaHCO_3_, 0.85 g C_8_H_5_KO_4_, 0.73 g peptone from casein, 1.70 g NH_4_Cl, 0.90 g CO(NH_2_)_2_, 0.224 g K_2_HPO_4_, 0.0175 g NaCl, 0.01 Ca_2_Cl and 0.005 g MgSO_4_ (per litre of distilled water) [34]. All reagents were purchased from PANREAC (Panreac, Química SAU, Barcelona, Spain). The real digestate (RD) used in this study was supplied by Valladolid WWTP (Spain). This digestate was obtained from the anaerobic digestion of mixed sludge and on-site centrifuged prior to use to eliminate suspended solids. The concentration of NH_3_ in SD and RD averaged 605.8 ± 0.1 and 678.9 ± 0.6 ppm, respectively, while pHs averaged 7.57 ± 0.05 and 8.99 ± 0.08, respectively.

### 2.2. Experimental Setup

**Figure 1:**
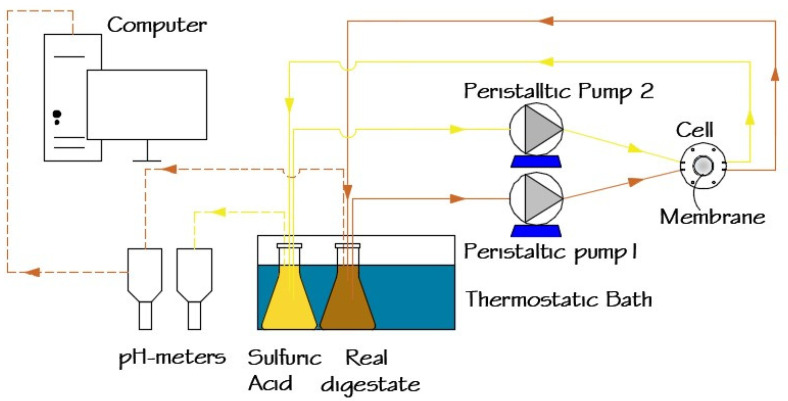
Schematic representation of the lab scale ammonia recovery system.

A schematic representation of the experimental setup used for ammonia recovery from digestates is shown in Figure 1. The target digestate (SD or RD) was continuously circulated using a peristaltic pump (Watson–Marlow Sci-Q 323, Spirax–Sarco Engineering plc, Cheltenham, England, UK) over the active layer of the membrane in a customized cell module [35]. A sulfuric acid solution was recirculated using a similar peristaltic pump on the support layer of the membrane. Both digestate and sulfuric acid solutions were maintained at 35 °C in a thermostatic bath (HAAKE type E12, Thermo Fisher Scientific, Waltham, MA, USA) in 0.5 L enclosed Erlenmeyer bottles.

The membranes used and their main characteristics are shown in Table 1. Polytetrafluoroethylene (PTFE) and polyvinylidene difluoride (PVDF) were selected as model commercial membranes based on their ideal properties for the separation of light molecules from water in a gaseous phase.

### 2.3. Operational Conditions and Process Evaluation 

An initial test series was carried out with all the membranes initially selected (Table 1) using synthetic digestate at two different pHs (7.6 and 10) and two sulfuric acid concentrations (0.005 and 1 M) at 35 °C with recirculation flow rates of 0.030 m^3^ h^−1^. Trials were carried out with intermediate sulfuric acid concentration, but with no relevance in the results. A second series of experiments was conducted with PTFE-0.22 and real digestate at pHs of 8.9 and 10 using recirculation flow rates of 0.030 m^3^ h^−1^ and a sulfuric acid concentration of 1 M. Finally, a third series of experiments was carried out with both RD and SD and the PTFE-0.22 membrane, at a pH of 10, with a sulfuric acid concentration of 1 M and recirculation flow rates of 0.007, 0.015, 0.030 and 0.045 m^3^ h^−^^1^. The membrane system was washed twice for 1 h after each experiment with distilled water when using SD, and with tap water (which contains sodium hypochlorite) when using RD. Samples of the digestate solution were drawn every 30 min over 3.5 h to analyse the concentration of NH_3_ and obtain pH values. Finally, a systematic evaluation of membrane fouling was performed by determining the pore size distribution of PTFE-0.22 membranes after their use with SD and RD, at a pH of 10 and with a sulfuric acid concentration of 1 M at different recirculation flow rates (0.007, 0.015, 0.030 and 0.045 m^3^ h^−1^). All experiments were carried out in duplicate. 

### 2.4. Analytical Methods 

The values of pH and temperature were monitored using a HI5522 pH meter Hanna Instruments (Woonsocket, RI, USA) and a Basic 20 pH meter (Crison Instruments, S.A., Alella, Barcelona, Spain) in the liquids on both sides of the membrane. NH_3_ was measured using the Nessler analytical method in a Shimadzu UV-160 A spectrophotometer at 425 nm (Shimadzu, Kyoto, Japan). 

### 2.5. Membrane Characterization Techniques 

Surface morphology was analysed by using Atomic Force Microscopy. Images were obtained with a Nanoscope IIIA microscope (Digital Instruments, Veeco Metrology Group, Chadds Ford, PA, USA) using the Tapping mode. Pore size distribution was analysed by the extended bubble point method, or Air Displacement Porometry (ADP), using a Coulter ^®^ Porometer-II manufactured by Coulter Electronics (Porometer, Aptco Invest, Dulles, VA, USA) [36]. Samples were first wetted with an electronic liquid FC-43 (Fluorinert^TM^, 3 M, St. Paul, MN, USA) of low surface tension (γ 1.6 × 10^−2^ N m^−1^), low vapour pressure (192 Pa) and low reactivity, that can be assumed to fill all the pores given a zero contact-angle with the membrane material. The wetted sample was subjected to increasing pressure, applied by a compressed clean and dry air source. The pressure range for PTFE 0.22 membrane was 0.09–0.60 µm. 

### 2.6. Theoretical Methods

The overall mass transfer coefficient of NH_3_ was calculated with Equation (2). according to [21].

(2) $$\frac{1}{k_{ov}}=\frac{1}{k_{s}}+\frac{1}{k_{m}}$$

where *k_s_* and *k_m_* are the mass transfer coefficients in the digestate side and within pores, respectively. The resistance on the acid solution side can be considered negligible (i.e., mass transfer coefficient on the acid side is much larger than *k_s_* and *k_m_*). In this context, the mass transfer coefficient on the digestate side can be estimated using Equation (3):

(3) $$k_{s}=\frac{D_{AW}Sh}{D_{H}}$$

where *D_AW_*, *Sh* and *D_H_* are: diffusion coefficient of NH_3_ in water (calculated using the software ASPEN (AspenTech, Bedford, MA, USA) at 35 °C), the Sherwood dimensionless number [37], and the hydraulic diameter, respectively. Similarly, the mass transfer coefficient within pores can be calculated by Equation (4) [26,38]:

(4) $$k_{s}=\frac{\epsilon D_{ij}}{\tau \delta }$$

where τ, δ, *D_ij_* and ϵ are: the tortuosity, the wall thickness of the membrane, the diffusion coefficient of NH_3_ in the air gap within pores, and the porosity of the membrane, respectively. Accepting Knudsen regime [21]:

(5) $$D_{ij}=\frac{d_{p}}{3}\sqrt{\frac{8RT}{\pi m}}$$

in terms of the pore diameter and NH_3_ molar mass m.

The molar theoretical flux of NH_3_ across the membrane can be estimated using Equation (6) [39]:

(6) $$J_{T}=k_{ov}\Delta C$$

here, ΔC is the difference in NH_3_ concentrations between the feed and permeate sides. Finally, NH_3_ recovery seems to follow first-order kinetics, which can be estimated using Equation (7) [40,41]:

(7) $$ln\frac{C_{0}}{C_{t}}=k_{ov}\frac{A_{m}}{V_{t}}t$$

where *k_ov_*, *A_m_*/*V_t_* and *t* are the overall mass transfer coefficient, the effective ratio of membrane area to feed volume of the digestate, and the elapsed time, respectively. *C*_0_ and *C_t_* are the concentration of ammonia at time zero and time *t* in the digestate, respectively. NH_3_ recovery (*R*) ranges between 0 and 1 (Equation (8)):

(8) $$R=1-\frac{C_{t}}{C_{0}}=1-e^{-k_{ov}\frac{A_{m}}{V_{t}}t}\equiv 1-e^{-\alpha t}$$

here $\alpha =k_{ov}\frac{A_{m}}{V_{t}}$ is the rate of concentration decrease and recovery increase. Therefore, the time needed to reach a given *R* can be estimated using Equation (9):

(9) $$t\left(R\right)=-\frac{ln\left(1-R\right)}{k_{ov}\frac{A_{m}}{V_{t}}}=-\frac{ln\left(1-R\right)}{\alpha }$$

## 3. Results and Discussion

### 3.1. Influence of Membrane Material, pH and H_2_SO_4_ Concentration 

**Figure 2:**
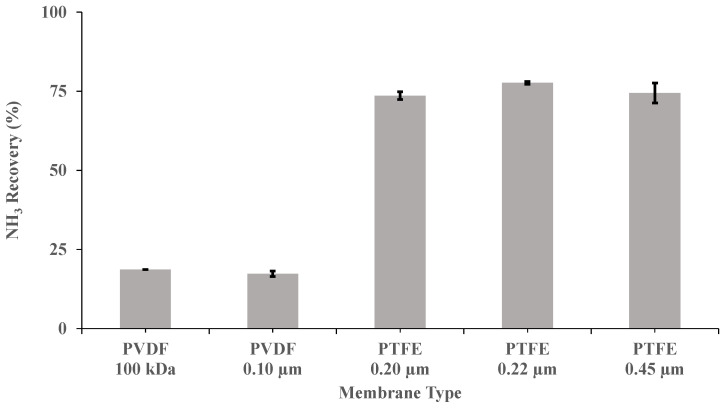
Influence of the type of membrane on ammonia recovery, after 3.5 h, in SD with a recirculation flow rate of 0.030 m^3^ h^−1^, a pH of 10 and a concentration of H_2_SO_4_ of 1 M. Vertical bars represent the standard deviation obtained from duplicate measurements.

Five flat sheet membranes (Table 1) were investigated at a recirculation flow rate of 0.030 m^3^ h^−1^, a pH of 10 and a concentration of H_2_SO_4_ of 1 M, to elucidate the optimum material, pore size and wettability for NH_3_ recovery from a synthetic digestate. PTFE membranes exhibited a superior performance to PVDF membranes in terms of NH_3_ recovery at a pH of 10 (Figure 2). In this case, the highest NH_3_ removal recovery after 3.5 h (77.7% ± 0.4%) was provided by PTFE-0.22. This removal efficiency was 3.6-fold higher than those obtained for the two PVDF membranes tested. As shown in Table 1, PTFE-0.22 has a contact angle (θ) of 150 degrees [42], which corresponds to a rather hydrophobic surface [43]. In fact, a membrane with θ > 140° could be considered super-hydrophobic according to Tylkowski et al. [43]. The superior performance of the PTFE-0.22 membrane could be also explained by its asymmetric structure, as membranes with symmetric structures, with similar thickness, have been consistently shown to be less permeable [20]. Therefore, the following experiments were also conducted using PTFE-0.22 membranes.

The influence of the digestate pH was also studied. This parameter determines NH_3_ mass transfer as it shifts the equilibrium ${\mathrm{NH}}_{4}^{+}\;↔{\mathrm{NH}}_{3\;}+H^{+}$. In our study, higher ammonia recoveries were achieved by increasing the pH of the synthetic digestate because of the increase in the NH_3_ concentration gradient (Figure S1 in the Supplementary material). Nevertheless, an increase in the pH of the digestate above 10 does not entail large improvement in the ammonia removal [20]. Operating the process at a pH of 11 with a PTFE-0.22 membrane, a recirculation flow rate of 0.030 m^3^ h^−1^ and a concentration of H_2_SO_4_ of 1 M, resulted in a NH_3_ recovery of 82.8% ± 0.4%, only 10% higher than the recoveries recorded at a pH of 10. NH_3_ recovery was lower in real digestate than in synthetic digestate using a PTFE-0.22 membrane at pHs of 10 and 7.6, the latter pH supporting lower NH_3_ recoveries (Figure S1 in the Supplementary material). Thus, the times needed to reach 95% NH_3_ recovery significantly increased by a factor of 6.5 and 2.3 in SD and RD, respectively, when digestate pH was increased from 7.6 to 10 (Table 2).

**Figure 3:**
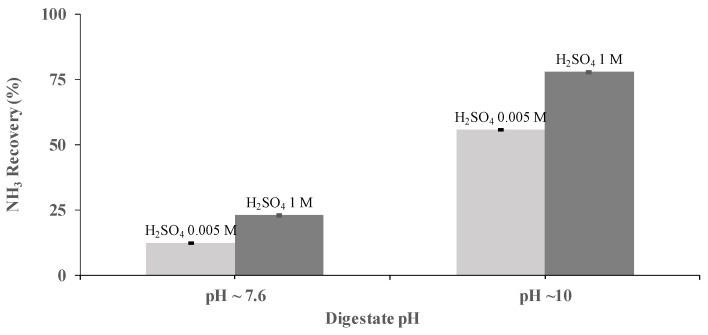
Influence of the sulfuric acid concentration on NH_3_ recovery from SD at pHs of 7.6 and 10 and a flow rate of 0.030 m^3^ h^−1^ using the PTFE-0.22 membrane. Vertical bars (very short here) represent the standard deviation from replicate measurements.

H_2_SO_4_ concentration was identified, also, as a key operational parameter for NH_3_ recovery. The performance of a PTFE-0.22 membrane at two concentrations of H_2_SO_4_ (0.005 and 1 M) was studied at pHs of 7.6 and 10, under a flow rate of 0.030 m^3^ h^−1^ using synthetic digestate. Acid concentrations of 0.005 M resulted in NH_3_ recoveries of 12.2% ± 0.2% and 55.5% ± 0.1% at a pH of 7.6 and 10, respectively, after 3.5 h of operation at the same flow rate. Similarly, under the same conditions, NH_3_ recoveries of 22.9% ± 0.4% and 77.7% ± 0.4% were achieved using H_2_SO_4_ concentrations of 1 M (Figure 3). Therefore, it was concluded that the best operating conditions correspond to a pH of 10 and H_2_SO_4_ concentrations of 1 M.

### 3.2. Influence of the Digestate Recirculation Flow Rate 

Ammonia removal efficiencies of 84.6% ± 1.0% were achieved using the PTFE-0.22 membrane after 3.5 h of experiment in synthetic digestate, which were higher than the removal efficiencies in real digestate (71.6% ± 0.3%), at 0.045 m^3^ h^−1^ and a pH of 10. The lower NH_3_ recoveries in real digestate were likely due to membrane fouling. This fouling affected the NH_3_ permeate flux mainly due to adsorption of colloids, or solutes including ammonia, within the membrane pores, with a subsequent reduction in mean pore sizes. According to Meng et al. [44], membrane fouling typically causes cake layer formation, deposition of sludge flocs and their deposition on the membrane. The influence of the velocity of the acid solution was not herein assessed as previous literature has consistently shown that the velocity of the acid solutions does not impact significantly on NH_3_ mass transfer [20,31]. 

**Figure 4:**
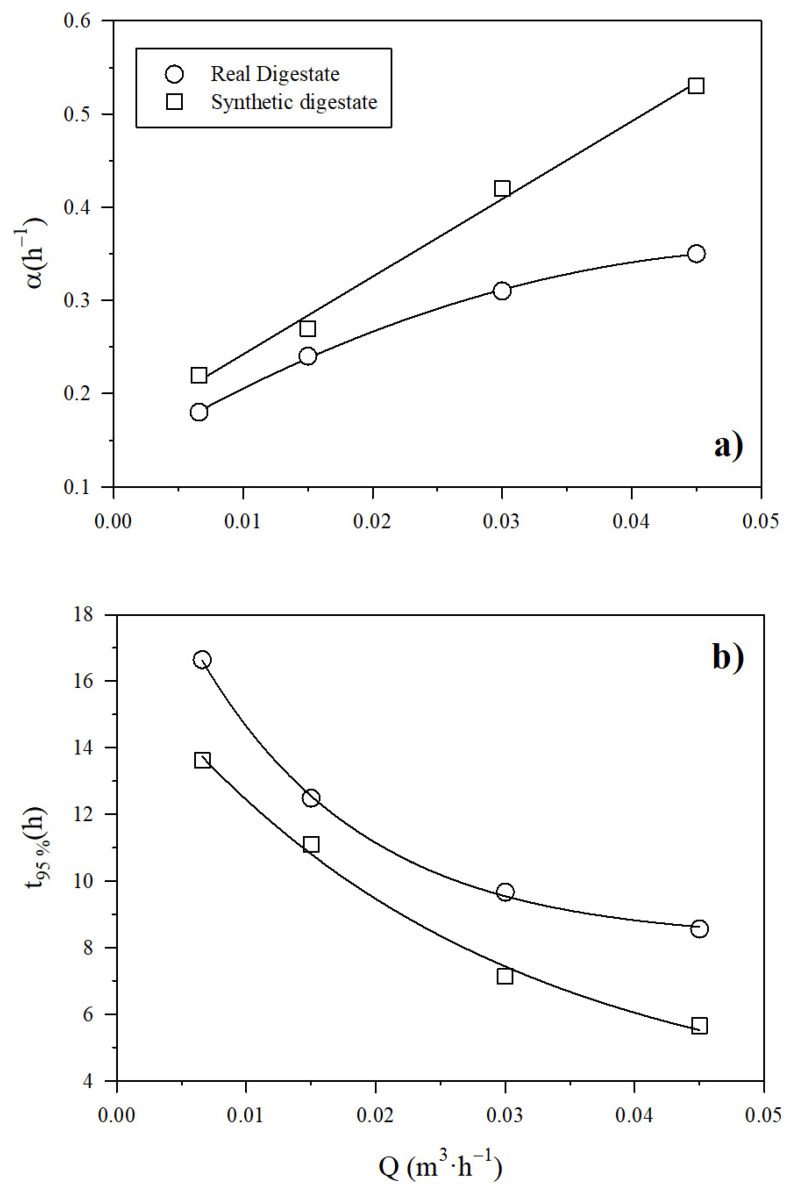
Influence of the recirculation flow rate on (**a**) α, and (**b**) the time to reach 95% NH_3_ recovery using a PTFE-0.22 membrane at different recirculation flow rates at a pH of 10 and a concentration of H_2_SO_4_ of 1M for both digestates.

The increase in the digestate recirculation flow rate brought about a rise in the pace of the NH_3_ recovery across the PTFE-0.22 membrane regardless of the type of digestate (real or synthetic). Figure S2a,b (in Supplementary Material) show the exponential decrease of the ammonia concentration, which resulted in a rapid increase of NH_3_ recovery (Equation (7)). In Figure 4a, the corresponding rate of concentration decrease and recovery increase α, is shown as a function of the recirculation flow rate of SD and RD (Equation (8)). The value of α was higher for the synthetic digestate, while the increase in digestate recirculation flow rate was positively correlated with NH_3_ recovery rate regardless of the type of digestate. Figure 4b shows the evolution of the time needed to reach 95% recovery (Equation (9)). This time is higher for the real digestate than for the synthetic one. It is worth noting that an increase of the recirculation flow of the digestate from 0.007 to 0.045 m^3^ h^−1^ brought about a substantial decrease of the time needed to reach 95% recovery of NH_3_. In all cases, a pH of 10 was maintained. The duration of the experiment conducted at a liquid recirculation rate of 0.03 m^3^ h^−1^ was extended to 6 h, in order to check how a longer time improved recovery. This experiment resulted in a final NH_3_ recovery of 94.3% ± 0.3% in synthetic digestate in accordance with Figure 4b. Therefore, we concluded that over long time intervals, the evolution of recovery follows the pace assumed here.

**Figure 5:**
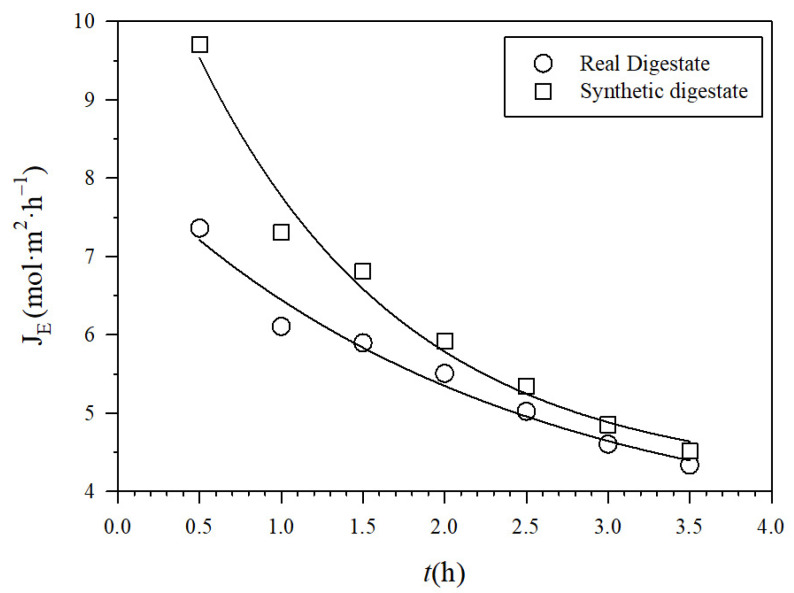
Experimental molar fluxes J_E_ for recirculation rate 0.030 m^3^ h^−1^ for PTFE-0.22 at a pH of 10 and 1 M H_2_SO_4_.

The NH_3_ fluxes across the PTFE-0.22 membrane in both SD and RD are shown in Figure 5. They decreased as the recirculation flow rate increased because of fouling, probably induced by shear. In addition, NH_3_ fluxes were slightly higher in SD than in RD according to its lower induction of membrane fouling. By fitting theoretical to experimental fluxes and using Equations (2)–(6), the corresponding effective pore diameters were evaluated at the end of each experiment, as a function of the recirculation flow Q, which was 0.25 µm. 

The experimental NH_3_ fluxes herein obtained with flat plate membranes were comparable to, but clearly higher than, those reported in the literature when using similar pHs with hollow fibre or tubular membranes (Table 3). 

In contrast, the overall mass transfer coefficient increased at increasing digestate recirculation flow rates. An analysis of the relative importance of the membrane mass transfer coefficient *k_m_*, and that on the digestate side *k_s_*, compared to the overall mass transfer coefficient *k_ov_*, show that the largest restriction to mass transfer occurred on the digestate side of the membrane, while through the pores, there was a very high NH_3_ mass transfer. NH_3_ mass transfer through the pores decreased steeply when the recirculation flow rate was increased, but with a concomitant increase of the mass transfer on the digestate side of the membrane. Values of Table 4 correspond to synthetic digestate.

### 3.3. Pore Size

The characteristics of the PTFE-0.22 membrane were studied by air displacement porometry after the operation of the process under different experimental conditions (pH of 10, concentration of H_2_SO_4_ 1 M, recirculation flow rates 0.007, 0.015, 0.030 and 0.045 m^3^ h^−1^) using both synthetic digestate and real digestate. The increase in membrane fouling reduced the pore diameter, as a result of deposits that partially or totally blocked the membrane pores (Table 5) [47]. A higher reduction in pore diameter was observed when the PTFE-0.22 membrane was exposed to longer periods of filtration, lower recirculation rates and real digestate from Valladolid WWTP. Interestingly, the mean pore size was similar in the new membrane and in the membranes used twice with recirculation flow rates of 0.045 m^3^ h^−1^ (0.3548 µm ± 0.0004 and 0.3521 µm ± 0.0004, respectively). This behaviour is in accordance with more specific fouling studies for PTFE in literature [48,49].

### 3.4. Membrane Morphology Analysis

**Figure 6:**
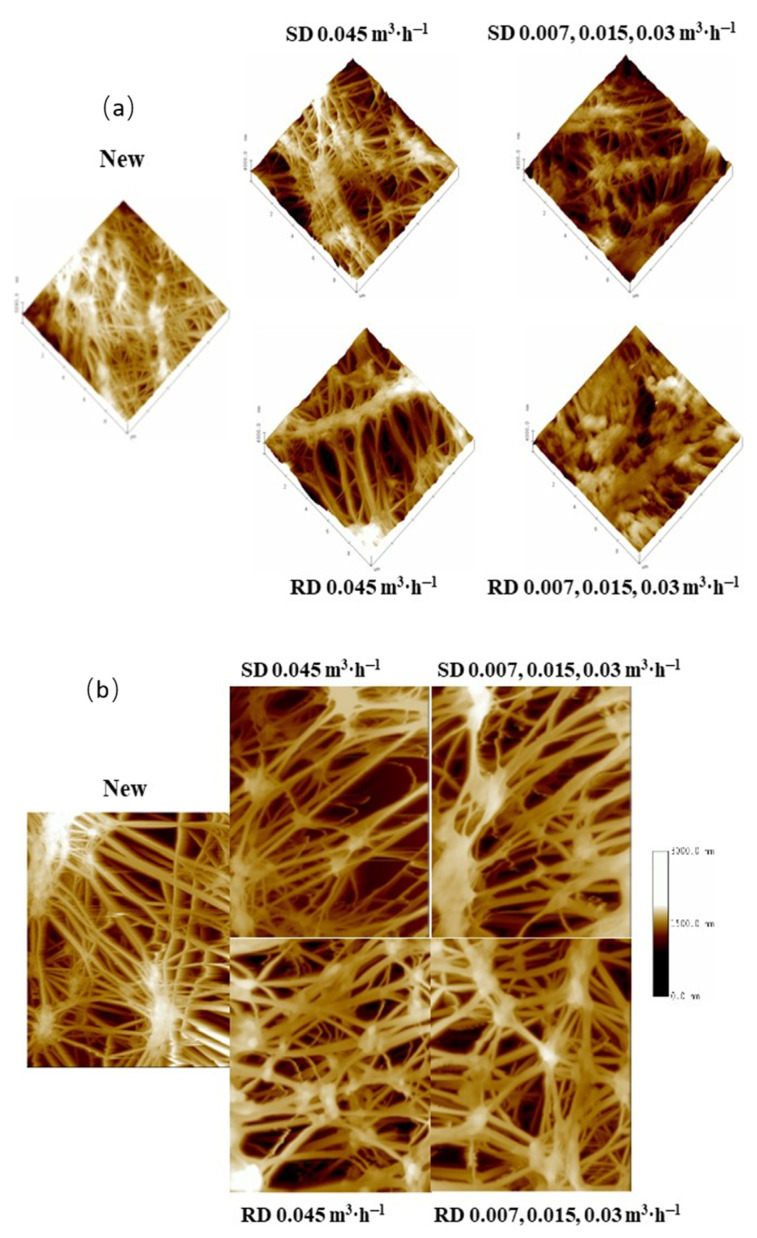
AFM 3D topographic images of the active layer (**a**) and 2D topographic images of the support layer (**b**) for the PTFE 0.22 µm membrane under different conditions (scanned area 10 µm × 10 µm). Legends correspond to the digestate, SD or RD, treated and the recirculation flow rates used successively.

Figure 6a shows three-dimensional AFM pictures of PTFE-0.22 membranes that were unused or used under multiple operational conditions. More fouling was detected on the surfaces of the membrane operated with real digestate at three recirculation flow rates. The membranes operated with real digestate at the highest circulation flow rate presented a surface fouling level in agreement with the pore size results (Table 4). The unused membrane has a topography with higher roughness than for the used membranes, in accordance with Zhang et al. [50].

In addition, the AFM topographic images of the support layer had a very similar appearance in all membranes tested, which suggested that the use of H_2_SO_4_ solutions did not significantly deteriorate the membrane surface (Figure 6b).

In addition, phase contrast images were obtained to detect the presence of materials coating the membrane surface (Figure S3 in Supplementary Material). The deeper brown tones detected in the membrane, used with real digestate and at three recirculation flow rates, would correspond to organic matter from the real digestate. Fouling was not significant in the rest of the samples and the coating effect was more homogeneous.

## 4. Conclusions

The feasibility of several commercial flat sheet membranes for ammonia recovery and their fouling were studied using synthetic and real digestate. PTFE was identified as the most efficient material for NH_3_ recovery regardless of the type of digestate. The highest NH_3_ recovery was obtained when the pH of the digestate was increased to 10 and 1 M sulfuric acid was used. NH_3_ recovery also increased at higher digestate circulation flow rates because of the inherent reduction in mass transfer resistance. NH_3_ recovery was shown to follow first order kinetics and to be faster under alkaline pH, high H_2_SO_4_ concentration and high recirculation flow rate on the digestate side of the membrane. A PTFE-0.22 membrane operated under optimal conditions supported NH_3_ recoveries of 71.6% ± 0.3% and 84.6% ± 1.0% after only 3.5 h using real and synthetic digestates, respectively. After 6 h of operation, 94.3% ± 0.3% NH_3_ recoveries were reached in synthetic digestate under similar operational conditions in accordance with the kinetics assumed here. Membrane fouling was relevant when using real digestate and resulted in a decrease in membrane pore diameter and growing surface deposition. 

## Figures and Tables

**Table 1:** Description of the flat sheet membranes studied.

Membrane	Material	Pore Size	Nominal Thickness (µm)	Contact Angle (θ)	Porosity (%)	Wettability	Manufacturer
PVDF-100	PVDF	100 kDa	160	130–135	*	hydrophobic	KOCH
PVDF-0.10	PVDF	0.10 µm	130	130–135	*	hydrophobic	Sterlitech
PTFE-0.20	PTFE	0.20 µm	139	142	*	hydrophobic	Pall Gelman
PTFE-0.22	PTFE	0.22 µm	175	150	70	hydrophobic	Millipore
PTFE-0.45	PTFE	0.45 µm	135	155	*	hydrophobic	Pall Gelman

* Information not supplied by manufacturers.

**Table 2:** Time needed to reach 95% NH_3_ recovery for the PTFE-0.22 membrane operated with a concentration of H_2_SO_4_ of 1 M and a flow rate of 0.030 m^3^ h^−1^.

	Digestate	
	SD	RD
pH	*t* (95%)/h	
7.6	36.03	16.88
10	5.49	7.31

**Table 3:** Summary of fluxes using hydrophobic membranes.

Matrix	*C*_0_ (ppm)	pH	*T* (°C)	H_2_SO_4_ (mol L^−1^)	Flux (mol m^−2^ h^−1^)	Membrane Configuration	Reference
Water containing NH_3_	400	9–10	40	0.3	0.11	Hollow fibre (PP)	[21]
Simulated wastewater	120	10	25	-	0.18	Hollow fibre (PVDF)	[20]
Digested effluents	1554	8	25	1	0.06	Hollow fibre (PP)	[45]
Raw swine manure	2390	9	25	1	0.33	Tubular (PE)	[46]
Landfill leachate	1300	10	25	0.1	1.27	Hollow fibre (PP)	[37]
Synthetic Digestate	679	10	35	1	4.52	Flat sheet (PTFE)	This work

**Table 4:** Calculated mass transfer coefficients for synthetic digestate.

Recirculation Rate (m^3^ h^−1^)	*k_ov_* ^a^ (m h^−1^)	*k_s_* ^b^/*k_ov_* (m h^−1^)	*k_m_* ^c^/*k_ov_* (m h^−1^)
0.007	0.13	3.48	1.40
0.015	0.15	5.91	1.20
0.030	0.16	9.73	1.11
0.045	0.17	13.23	1.08

^a^ Calculated from Equation (2). ^b^ Calculated from Equation (3) *D*_aw_ = 1.26 × 10 ^−5^ m^2^ h^−1^. ^c^ Calculated from Equation (4) *D_ij_* = 0.26 m^2^ h^−1.^

**Table 5:** Mean pore sizes of the PTFE-0.22 membrane under different operational conditions.

Type of Digestate	Recirculation Rate (m^3^ h^−1^)	Type of Membrane	Mean Pore Size (µm)
None	None	New membrane	0.3548 ± 0.0004
SD	0.045	Used twice	0.3521 ± 0.0004
SD	0.007, 0.015, 0.030	Used multiple times	0.3443 ± 0.0004
RD	0.045	Used twice	0.3347 ± 0.0003
RD	0.007, 0.015, 0.030	Used multiple times	0.3228 ± 0.0002

## Data Availability

Not applicable.

## Supplementary Materials

The following are available online at https://www.mdpi.com/article/10.3390/membranes12010019/s1, Figure S1: Influence of the pH in (a) SD and (b) RD on the time course of NH_3_ concentration in the feed side of the PTFE-0.22 membrane operated with a concentration of H_2_SO_4_ of 1 M and a recirculation flow rate of 0.030 m^3^ h^−1^. Figure S2: Time course of ammonia recovery using a PTFE-0.22 membrane at different recirculation flow rates, a pH of 10, a concentration of H_2_SO_4_ of 1 M in (a) synthetic digestate and (b) real digestate. Figure S3: AFM Phase Imaging of the active layer for a PTFE 0.22 membrane under different conditions (scanned area 10 µm × 10 µm).
