# Supplementary material for: A Systematic Study of Ammonia Recovery from Anaerobic Digestate Using Membrane-Based Separation
Source: Membranes (Basel). 2021 Dec 24;12(1):19. doi: 10.3390/membranes12010019 (PMC8777830; doi:10.3390/membranes12010019)
Supplement: Supplementary file 1 [file membranes-12-00019-s001.zip › membranes-1525305-supplementary.pdf]

membranes

Article

A Systematic Study of Ammonia Recovery from Anaerobic Digestate Using Membrane-Based Separation

Fanny Rivera [1,2], Raúl Muñoz [1,3], Pedro Prádanos [1,2], Antonio Hernández [1,2] and Laura Palacio [1,2,*]

[1] Institute of Sustainable Processes, University of Valladolid, 47011 Valladolid, Spain; fannymaritza.rivera@uva.es (F.R); mutora@iq.uva.es (R.M.); ppradanos@uva.es (P.P.); antonio.hernandez@uva.es (A.H.)
[2] Department of Applied Physics, Science Faculty, University of Valladolid, 47011 Valladolid, Spain
[3] Department of Chemical Engineering and Environmental Technology, University of Valladolid, 47011 Valladolid, Spain
* Correspondence: laura.palacio@uva.es (L.P.)

Citation: Rivera, F.; Muñoz, R.; Prádanos, P.; Hernández, A.; Palacio, L. A Systematic Study of Ammonia Recovery from Anaerobic Digestate Using Membrane-Based Separation. *Membranes* **2022**, *12*, 19. https://doi.org/10.3390/membranes12010019

Academic Editors: Heitor Bento and Cristiano E. Rodrigues Reis

Received: 9 December 2021
Accepted: 23 December 2021
Published: 24 December 2021

Publisher's Note: MDPI stays neutral with regard to jurisdictional claims in published maps and institutional affiliations.

Copyright: © 2021 by the authors. Licensee MDPI, Basel, Switzerland. This article is an open access article distributed under the terms and conditions of the Creative Commons Attribution (CC BY) license (https://creativecommons.org/licenses/by/4.0/).

Supplementary Materials

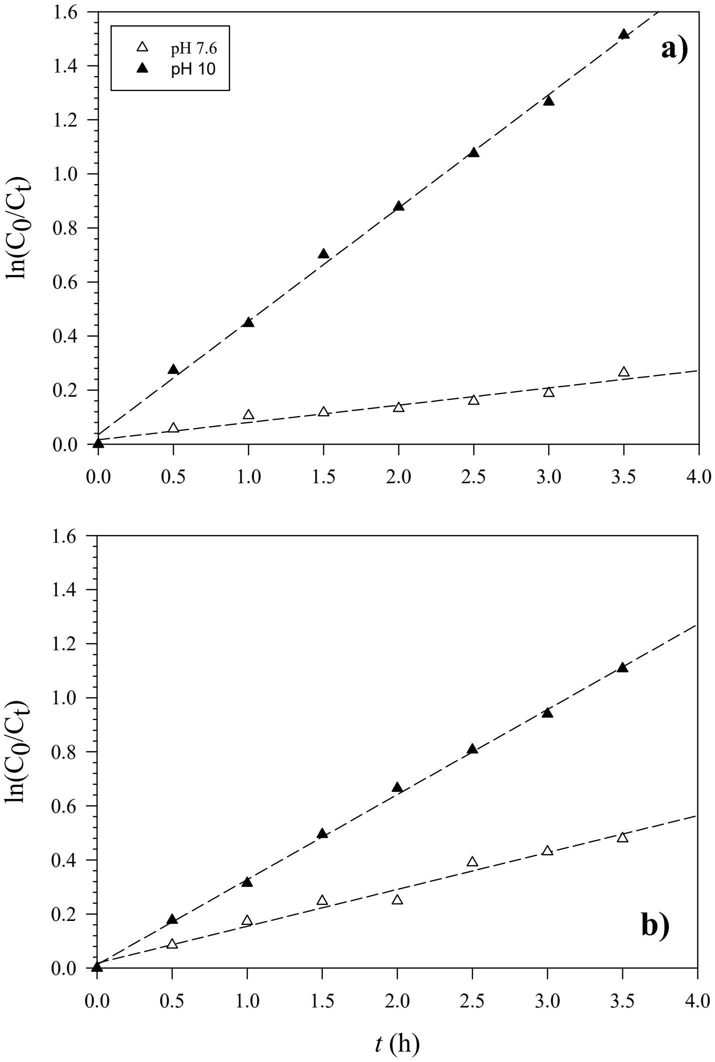

Figure S1. . Influence of the pH in a) SD and b) RD on the time course of NH_3 concentration in the feed side of the PTFE-0.22 membrane operated with a concentration of H_2SO_4 of 1M and a recircuN

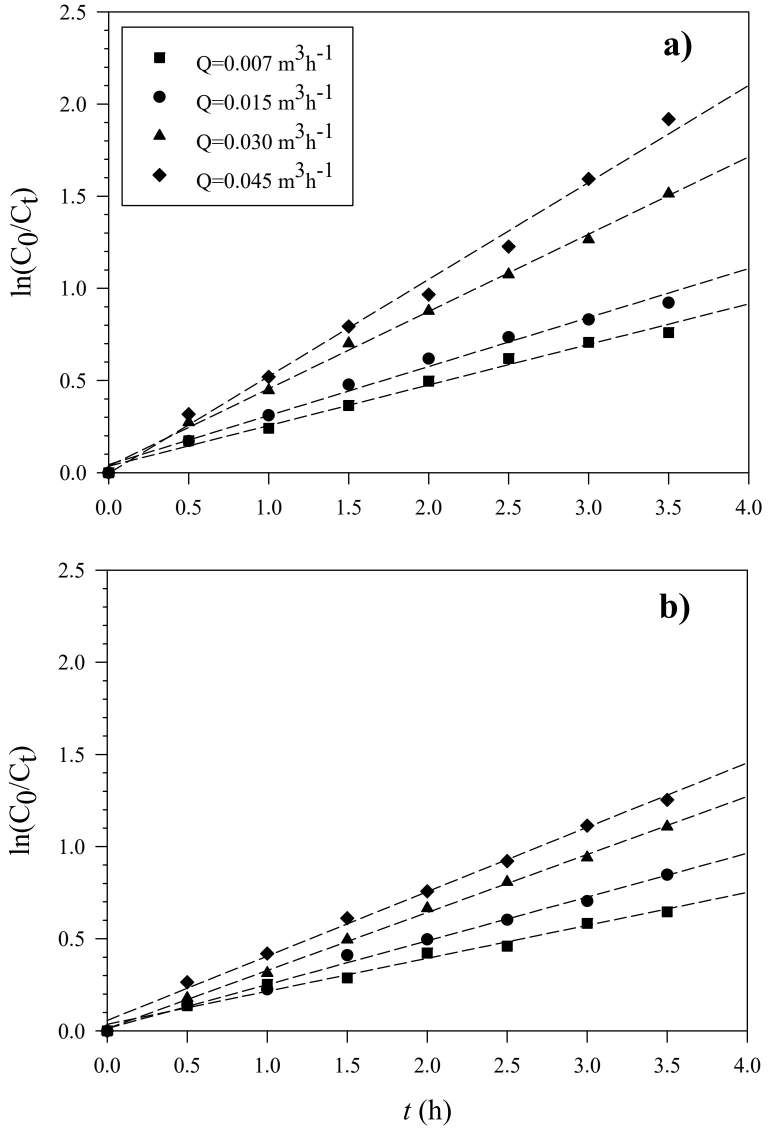

Figure S2. Time course of ammonia recovery using a PTFE-0.22 membrane at different recirculation flow rate, pH 10, concentration of H_2SO_4 of 1M in a) synthetic digestate and b) real digestate.

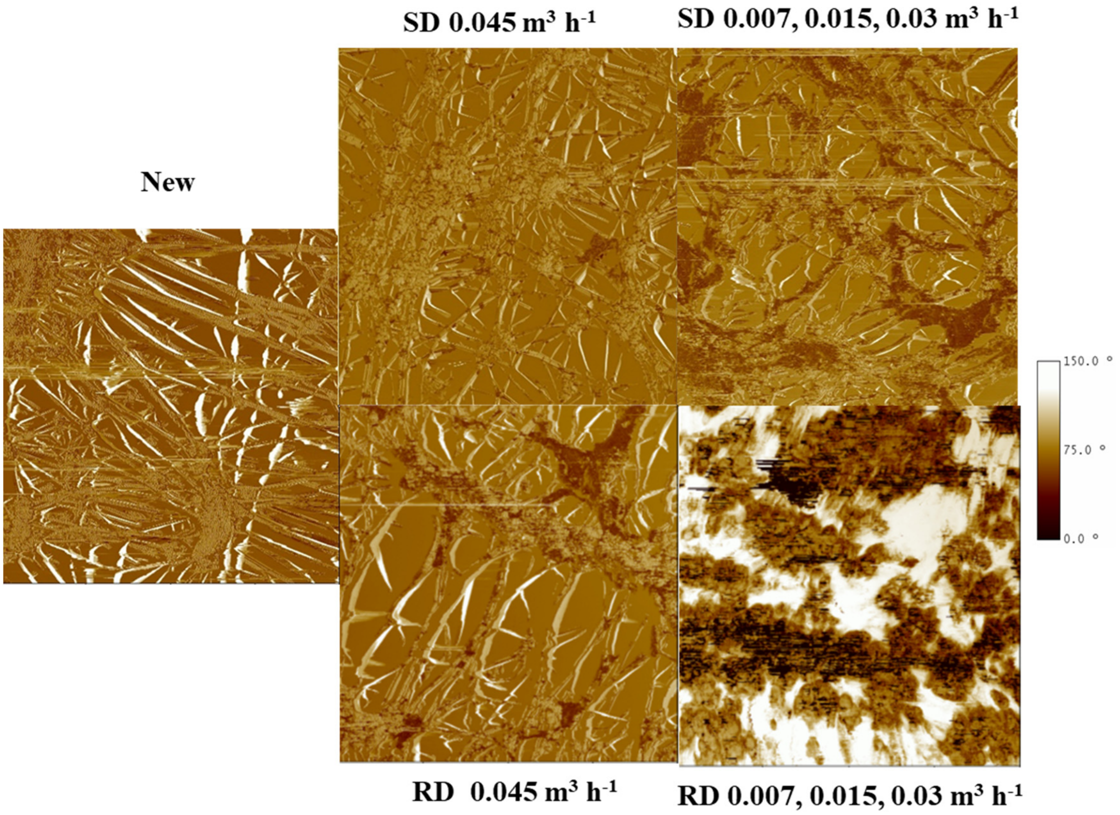

Figure S3. AFM Phase Imaging of the active layer for PTFE 0.22 membrane under different conditions (scanned area 10µm × 10 µm).
